# Neurophysiological study of consumer emotional reactions in a simulated multisensory retail environment

**DOI:** 10.3389/fnhum.2025.1635673

**Published:** 2025-10-24

**Authors:** Julia Eremenko, Vladimir Kosonogov, Vladislav Aksiotis, Victoria Moiseeva, Anastasia Obukhova, Alisa Godovanets, Oksana Zinchenko, Vasily Klucharev, Anna Shestakova

**Affiliations:** ^1^Centre for Cognition & Decision Making, Institute for Cognitive Neuroscience, National Research University Higher School of Economics, Moscow, Russia; ^2^Center for Bioelectric Interfaces, Institute for Cognitive Neuroscience, National Research University Higher School of Economics, Moscow, Russia; ^3^Phystech School of Biomedical and Medical Physics, Moscow Institute of Physics and Technology, Moscow, Russia; ^4^International Laboratory for Social Neurobiology, Institute for Cognitive Neuroscience, National Research University Higher School of Economics, Moscow, Russia

**Keywords:** retail space, multisensory integration, VR, heart rate variability, electrodermal activity

## Abstract

**Introduction:**

Emotions play a crucial role in shaping consumer experiences and decisions. Neurophysiological tools offer objective markers of emotional reactions in multisensory environments, where positive valence promotes approach behavior and negative valence fosters avoidance.

**Methods:**

We applied the Osgood semantic differential (SD) to establish correspondences between visual, auditory, and olfactory stimuli and target emotions relevant to retail zoning. Based on SD results, we selected stimuli to create multisensory environments. These were presented in immersive virtual reality (VR) to 27 participants. Emotional responses were assessed via heart rate (HR), heart rate variability (HRV), and electrodermal activity (EDA).

**Results:**

SD analysis identified cross-modal associations between sensory stimuli and retail zones, allowing refinement of semantic positioning. VR experiments revealed that HRV significantly increased in pleasant environments, indicating enhanced parasympathetic activation. HR and EDA showed no significant correlation with emotional valence, though both displayed trends toward reduction in pleasant conditions.

**Discussion:**

Our findings suggest that HRV is a reliable physiological marker of consumers’ approach behavior in multisensory retail environments, whereas HR and EDA are less sensitive. Combining SD with VR-based neurophysiological assessment enables objective evaluation of emotional zoning strategies, offering a scientifically grounded alternative to intuitive design practices for optimizing consumer experience.

## 1 Introduction

Current scientific findings in behavioral economics, neuromarketing, and consumer psychology point to the importance of emotions in shaping consumer experiences and decision-making (see Vences et al., 2020, for a review). In his book “*Emotional Design*,” Norman (2007) argues that a product’s consumer value is determined not only by its useful properties but also by the emotional component of consumer interactions. Norman further discusses the need to explore the emotions that consumers experience when making a purchase or using a product. This is a key concept in the marketing strategies of modern global brands and has led to the emergence of branches such as affective and sensory marketing, emotional design, and kansei engineering. The focus of the described fields is to design an emotional customer experience at all points of a brand’s interaction with customers (Brakus et al., 2009; Lemon and Verhoef, 2016).

The use of multisensory stimuli has become a natural way for an enterprise to communicate with its target audience (Abdolmohamad Sagha et al., 2022). It also allows for the emotional and cognitive zoning of a retail space in accordance with consumer preferences and expectations on the one hand and business objectives on the other. For example, a customer may want a sense of naturalness, lightness, and healthiness in the area of healthy foods; a feeling of joy, surprise, or delight in a kid’s toy store, and an atmosphere of calm, relaxation, and enjoyment near a cash register. However, in practice, the choice of a design concept or a set of sensory stimuli is based on personal intuition or willingness and the professional experience of owners, designers, and marketers, which can lead to inconsistencies and errors in the design of an emotional multisensory experience.

The atmosphere of a store has a direct impact on the overall formation of consumer experiences (Levy et al., 2004). It also influences the time and money spent by a customer in the store, the pace at which a purchase decision is made, and the probability of subsequent regret about a purchase (Jalil et al., 2016; Mohd-Ramly and Omar, 2017). Consequently, the choice of color, music, odor, or lighting for the sales floor is a marketing task requiring an objective scientific approach that would allow the business to achieve its goals and objectives.

When developing a new product or designing a retail space, entrepreneurs initially focused on physical and cognitive aspects, aiming to prevent stress, discomfort, fatigue, pain, injury, etc. From the 1990s, many marketing researchers began to promote the use of positive approaches, including flow experience (Csikszentmihalyi and Csikzentmihaly, 1990) and hedonomics (Helander and Po Tham, 2003; Hancock et al., 2005). These approaches are aimed at inducing pleasant emotions and impressions when customers interact with a product. The concept of emotional design (Norman, 2007) was conceived in line with these approaches. In his book “Emotional Design,” Donald A. Norman states that emotions are humans’ physiological responses to the roles of external things and themselves and are determined by their needs and expectations. The author’s approach to emotional design includes three stages: intuitive (a product’s appearance), behavioral (a product’s usability), and reflexive (personal satisfaction, self-esteem, and commemoration). Norman’s theoretical approach has been elaborated upon in the areas of affective computing and sensory marketing.

Ten years before, Nagamachi (1994) proposed the concept of kansei engineering (“kansei”—sensations, feelings), which involves designing emotional experiences for consumers through specific product features and design. The concept became popular after it was used by Mazda for the development of the MX-5 sports car, which entered the Guinness Book of Records as the most successful and best-selling two-seat sports car (Mazda Motor Corporation, 2011). The company’s success in the market contributed to the widespread application and further development of the concept. To understand which feelings and/or sensations (kansei) certain elements of product design evoke in consumers, Nagamachi (1994) suggested the use of behaviors, body expressions, and the semantic differential (SD) as screening instruments. The SD approach involves evaluating a concept on a scale, the poles of which are represented by antonym adjectives, such as “expensive–cheap,” “original–typical,” and “big–small,” etc. This approach has received enormous attention from psychologists and social and behavioral scientists (Rosenberg and Navarro, 2018) since its invention by Osgood (1964).

In the 1990s, sensory marketing began to expand. This approach refers to modulating consumers’ emotional states using sensory stimuli to further influence consumer behavior and experience. The topic of sensory marketing was first addressed by Kotler (1973), one of the pioneers of marketing research. He proposed the concept of “store atmosphere,” referring to the deliberate design of retail spaces to elicit specific cognitive or emotional reactions among consumers. Krishna (2012) defined it as a tool that can influence the consumers’ feelings and, in turn, their perceptions, judgments, and behaviors. He argued that sensory stimuli can act as unconscious triggers that trigger consumers’ perceptions of abstract product concepts, such as reliable, refined, or poetic, thereby attracting their attention and facilitating effective communication. In line with Krishna’s and Kotler’s views, Bindu et al. (2021) elaborated on the influence of a store’s internal environment on purchasing behavior. According to Mehrabian and Russell (1974) theory, an environment has certain stimuli (S) that lead to changes in an organism (O), and these changes cause a certain reaction of the recipient (R), such as approaching or avoiding. The authors attempted to explain individuals’ emotional reactions in response to certain stimuli in external environments; they described the structure of emotions using a P-A-D model, which consisting of pleasure/displeasure (P), arousal/passivity (A), and dominance/submissiveness (D). A positive emotional reaction in response to multisensory stimuli leads to desired business outcomes (Abdolmohamad Sagha et al., 2022). Fundamental psychophysiological studies of emotions have greatly impacted sensory neuromarketing and have linked neuromarketing results with consumer neuroscience and the classical theory of emotions and motivation. Harmon-Jones et al. (2010b) suggested that, in most cases, positive and negative emotions can be correlated with corresponding motivational approaches such as approach or avoidance, though not without rare exceptions, such as when anger, a negative emotion, is accompanied by approach motivation (Carver and Harmon-Jones, 2009). Carver and Harmon-Jones (2009) highlighted that anger implies the failure to achieve a desired state or goal, leading to approach motivation. Croy et al. (2013) associated unpleasant emotions, even disgust, with an avoidance response aimed at protecting the body from harmful influences. The application of the motivational approach (Harmon-Jones et al., 2010a) can serve as a framework for assessing consumers’ emotions and feelings in the retail space, where they are expected to exhibit approach and avoidance behaviors for pleasant and unpleasant sensory stimuli, respectively.

### 1.1 Sensory stimuli in modeling consumer emotional and behavioral responses

A considerable amount of evidence has been gathered regarding the effects of different sensory stimuli on people’s emotional states. The most frequently used sensory stimuli are color, music, and scent.

#### 1.1.1 Color

In the organization of retail spaces, product and packaging design, and the development of promotional materials, color is the most sought-after and studied factor. A number of studies (Adams and Osgood, 1973; Greene et al., 1983; Hupka et al., 1997; Kaya and Epps, 2004; Heller, 1990) have revealed the influence of color on emotions. In these and other experiments, color characteristics were manipulated to evoke specific emotional reactions to video content or advertising. For example, Wilms and Oberfeld (2018) showed that hue, saturation, and brightness can significantly impact the valence and arousal of emotions. They found that saturation and brightness increased the level of excitation and valence and that changing the hue from blue and green to red led to a significant increase in excitation. Studies suggest a complex relationship between lighting and customer’s emotional state (Park and Farr, 2007). Some articles (Gao et al., 2007; Suk and Irtel, 2010; Valdez and Mehrabian, 1994) have shown that color saturation has a greater influence on human emotions than hue.

Color plays a pivotal role in influencing consumer behavior, affecting brand perceptions, purchasing decisions, and cognitive processes. Rapid product evaluations rely heavily on color, with consumers making judgments within 90 s, and color accounting for 62 to 90% of these assessments (Singh, 2006). This makes color a crucial marketing stimulus that operates through both conscious and subconscious mechanisms. The color-in-context theory conceptualizes how color triggers psychological functioning via both innate and learned pathways (Elliot, 2015). Specifically, the red color tends to activate avoidance motivation and improves detail-oriented task performance, whereas the blue color facilitates approach motivation and enhances creativity (Elliot, 2015). These cognitive effects extend to marketing contexts, where warm-colored advertisements can increase visual attention and generate favorable implicit attitudes compared to cool colors (Labrecque and Milne, 2012). Color also functions as a key element in conveying brand personality, with empirical evidence linking red to excitement, blue to competence and trust, and green to sincerity and environmental awareness (Labrecque and Milne, 2013).

#### 1.1.2 Music

Music affects the cognitive processes and emotional states of consumers, consequently influencing all stages of consumer decision-making: awareness of need, search for information, evaluation of alternatives, purchase, and post-purchase state. Music characteristics such as tempo, melody, volume, rhythm, and harmony can evoke a spectrum of different emotions (Zhu and Meyers-Levy, 2005), mediating the modulatory effect of the music on behavioral markers. For example, some studies have revealed that auditory stimuli can affect the time customers spends in a store and the amount of money they spend on purchases (Yalch and Spangenberg, 2000; Caldwell and Hibbert, 2002). Other studies have revealed the effects of music on customers’ overall assessment of a store’s atmosphere (Spangenberg et al., 2005), overall shopping experience (Garlin and Owen, 2006), and level of consumption (Stroebele and de Castro, 2006).

#### 1.1.3 Odor

In contrast to research on color and music, odor remains poorly understood because of the methods available to assess and measure it. For instance, there are no approaches for assessing absorption spectra and describing odors equivalent to those for color perception. In addition, odor perception is significantly influenced not only by the physiological characteristics of the stimulus but also by psychological characteristics, such as prior experience, affective state, and contextual expectations, which complicates the acquisition of objective data (Herz, 2016; Spence, 2020; Dalton, 2000). The role of odors in the commercial sphere is increasing every year due to their influence on various cognitive and affective processes (Spence, 2020; Herz, 2016; De Luca and Botelho, 2021). Many studies have described the effects of odors on brain function, as scents are able to cross the blood–brain barrier and interact with receptors in the central nervous system (Kutlu et al., 2008; Touhara and Vosshall, 2009). Emotional and behavioral responses to various olfactory cues have been widely studied using physiological methods such as electroencephalography (EEG), functional near-infrared spectroscopy (fNIRS), and magnetic resonance imaging (fMRI) (Angelucci et al., 2014; Grabenhorst et al., 2011; Sayorwan et al., 2012; Schulz et al., 1998; Iijima et al., 2009; Matsubara et al., 2011; Skorić et al., 2015).

The significant effects of odor on human psychophysiology have led to the emergence of several studies on sensory marketing, with scent considered an independent variable that can influence consumer behavior. A number of studies (Morrin and Ratneshwar, 2000; Orth and Bourrain, 2005) have shown the effects of odor on brand recall, risk-taking, diversity, and curiosity. In retail, the ability of olfactory stimuli to enhance emotion and reduce the perception of time spent has been shown in Leenders et al.’s (2019) study. Guéguen and Petr (2006) and Holland et al. (2005) reported that unconscious perceptions of fragrances can lead to changes in consumer behavior. For example, a citrus scent was found to increase the time one spent in a store and the number of purchases made. A review by Rimkute et al. (2016) on the influence of odor on purchasing behavior highlighted the importance of scents in modeling emotions and, consequently, on consumer behavior. In addition, the review showed the influence of olfactory sensory stimuli on the duration of the in-store selection process for a particular product, willingness to pay, amount of money spent, and time spent in the sales floor.

#### 1.1.4 Multisensory approach

Recent studies have evidenced the value of multisensory exposure in achieving marketing goals and objectives. For example, Duong et al. (2022) investigated how an increase in the number of stimulated sensory modalities can affect consumers’ emotional responses and, consequently, store image and brand attitude. Spangenberg et al. (2005) and Grewal et al. (2014) showed how a multisensory environment significantly enhances the emotional responses of shoppers in retail; congruence between the arousal levels of scent and music (both high or both low) led to positive consumer evaluations compared to incongruent conditions (e.g., high scent with low music).

From the abovementioned results, it is evident that a multisensory approach is beneficial in marketing research. However, some questions remain: How can an assortment of stimuli be selected objectively to modulate consumers’ cognition and behavior, and how can the preponderance of one sensory stimulus combination over another be evaluated? Neuroscience appears to provide solutions to these problems.

### 1.2 Neurophysiological measurements

A new field called consumer neuroscience or neuromarketing has largely benefited from the development of affordable wearable neurophysiological instruments such as portable EEGs, eye tracking, and polygraphic measurements of heart rate (HR) and electrodermal activity (EDA), which complement marketing research. Neurophysiological methods have several significant advantages compared to traditional marketing research because they prevent subjective bias (Balconi and Sansone, 2021; Pozharliev et al., 2017). HR and EDA monitors are among the most frequently used instruments for measuring psychophysiological reactions of consumers due to their low cost and high accessibility (both can be tracked using a wristband) (see Hickey et al., 2021 for a review). EDA and HR are signals of the autonomic nervous system, and they increase during arousal. More specifically, EDA measures skin conductance, which reflects sympathetic nervous system activity. It is often used to gauge emotional arousal, as higher EDA levels indicate greater arousal in response to stimuli (Kreibig et al., 2007). HR is influenced by both sympathetic and parasympathetic nervous system activity. Heart rate variability (HRV) provides insights into autonomic balance, with high-frequency components reflecting vagal tone and low-frequency components associated with sympathetic activation. Research has shown that positive emotions are linked with reduced HR and increased HRV, while negative emotions are often associated with increased HR and reduced HRV (Yu et al., 2024).

A number of studies have revealed the relationship between EDA, HR indicators, and the valence of stimuli (Kreibig, 2010; Shiota et al., 2011; Bradley and Lang, 2000). Bradley and Lang (2000) found that pleasant images result in lower EDA than unpleasant images, indicating a clear relationship between emotional valence and EDA. Similarly, emphasized that physiological responses measured through EDA and HR vary significantly depending on whether the associated stimuli are perceived as positive or negative. In pleasant conditions, HRV usually increases (Kumpulainen et al., 2024; Benz et al., 2022) because positive emotions and relaxation activate the parasympathetic nervous system. The autonomous system’s reactions are divided between the parasympathetic and sympathetic nervous system. The former is associated with rest and relaxation (e.g., decreased heart rate), while the latter is associated with the fight-or-flight response and increased arousal and attention (e.g., Pham et al., 2021; Waxenbaum et al., 2023).

Multiple studies have provided substantial evidence that sensory and affective factors play an important role in shaping consumer experiences (e.g., Herz, 2016; Spence, 2020). However, much of this work has relied primarily on self-reported data, which are prone to subjective biases and fail to capture implicit, unconscious processes. At the same time, neurophysiological research has advanced our understanding of consumer responses at a neural and emotional level, yet these insights remain insufficiently integrated with the broader framework of multisensory brand experience. This disconnect leaves an important gap: we lack a coherent understanding of how neurophysiological measures can concretely complement and extend traditional approaches to studying consumer experience.

To address this gap, our study investigates (1) how multisensory stimulation contributes to consumer experience and (2) how the combination of neurophysiological methods and VR provide unique insights compared to traditional measures. These questions directly respond to the need for greater theoretical and methodological integration, thereby advancing both academic understanding and practical application.

This psychophysiological approach was tested using the case of a luxury jewellery salon. For different areas of the retail space, it was necessary to select the most relevant set of sensory stimuli that corresponded to consumers’ emotional and cognitive needs as well as the business goals and objectives. For our study, we selected multisensory stimuli relevant to the retail environment and customer expectations, which when simultaneously presented could induce specific emotional and cognitive states in customers. To collect objective quantitative data on customers’ perceptions, we decided to combine SD measurements of consumer feelings and emotions with measurements of HR, HRV, and EDA. We first identified the correlation between sensory stimuli and the desired set of emotions using SD and then simulated multisensory environments in the lab to model real-market consumer behavior. The second stage was dedicated to testing the resulting multisensory environments for approach/avoidance effects using HR, HRV, and EDA. We created a number of multisensory spaces using stimuli from the first stage as well as immersive technology to demonstrate color in virtual rooms, headphones to present music, and odor samples.

## 2 Materials and methods

Our two-stage approach may be useful for multisensory zoning of commercial spaces. It involves two sequential stages. The first stage of the proposed approach involves obtaining a quantitative characterization of the target audience’s emotional attitudes toward sensory stimuli. For this purpose, the semantic differential (SD) technique is recommended. This approach entails rating a concept on a 7-point scale, the poles of which are represented by adjective antonyms, for example, “expensive–cheap,” “large–small.” The outcome of the first stage is the identification of correspondences between sensory stimuli and the list of concepts.

The second stage involves the application of a wide range of physiological methods to obtain objective data on the emotional and physiological responses of consumers situated in modulated multisensory environments. At this stage, the creation of relevant multisensory spaces is envisaged, using stimuli derived from the first stage, as well as immersive technologies to present color in virtual rooms, headphones for auditory stimuli, and scent samples. As previously mentioned, positive emotions are expected to facilitate an “approach” effect, whereas negative emotions may induce “avoidance” behavior.

### 2.1 Phase 1: SD test

During the first stage of the experiment, we established cross-modal correspondences between the sensory stimuli selected by the company and the desired set of sensations and emotions for each zone. For this, we used the SD procedure.

#### 2.1.1 Participants

A total of 46 people (25.6% men, 74.4% women; average age = 23.4 years; SD = 6.6) took part in the first stage. The participants were invited through public announcements. The study was conducted in compliance with the principles given in the Declaration of Helsinki, and all subjects provided written informed consent at the beginning of the study. The research protocol was approved by the ethics committee of the HSE University.

#### 2.1.2 Stimuli

**Figure 1:**
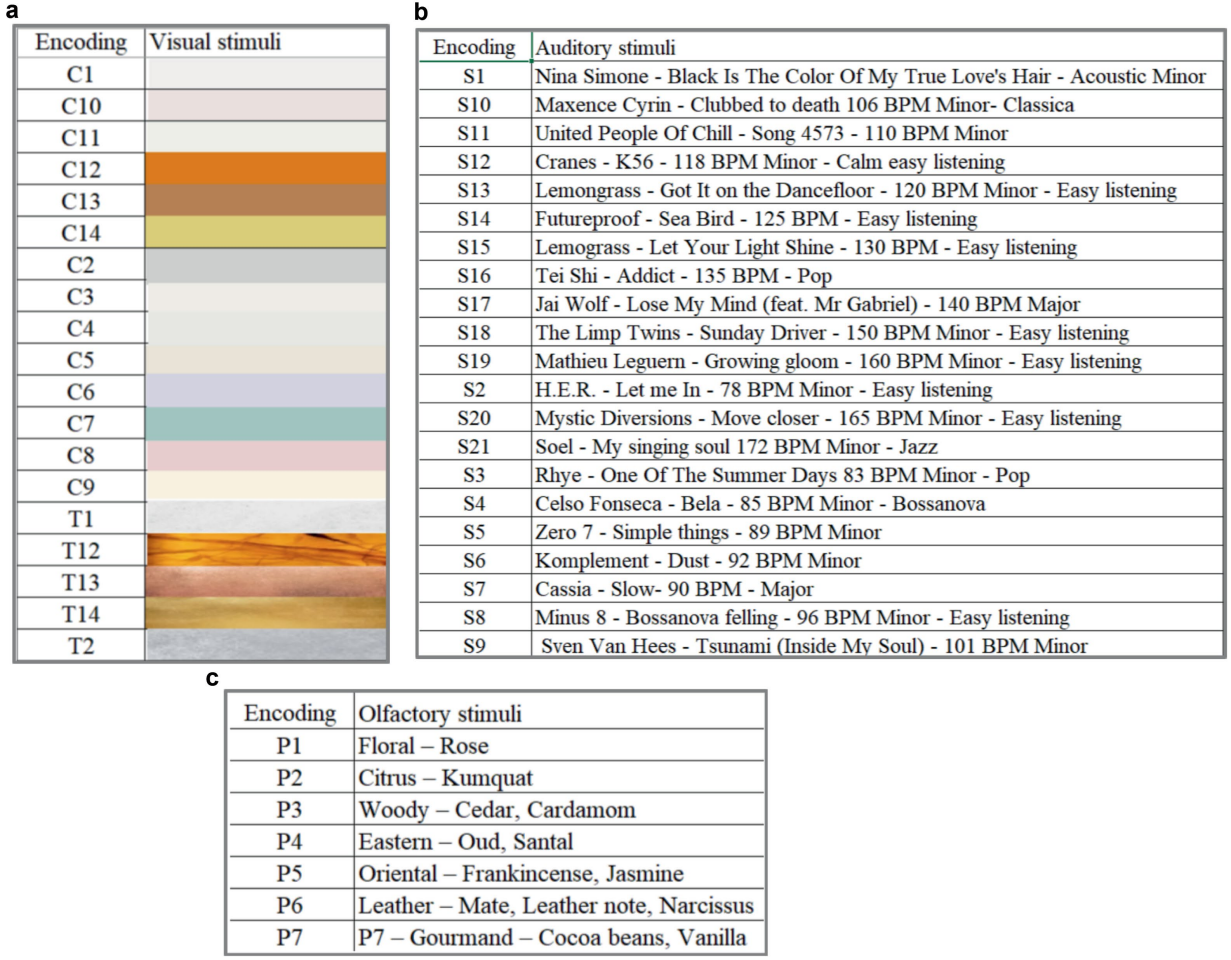
Corporate sensory incentives for testing **(a)** corporate visual sensory stimuli, **(b)** corporate auditory sensory stimuli, **(c)** corporate olfactory sensory stimuli.

Cartier Dom, a company that specializes in selling jewelry, provided descriptions of the zones in its retail space as follows: Welcome zone (inspiring, curious, majestic), Cashier zone (calm, private, caring, confident, effective, reliable), Wedding Rings zone (perfect, pure, eternal, ethereal, poetic), Diamond zone (passionate, bold, expressive, impressive). They also provided 21 pieces of music, 14 colors, 5 textures, and 7 odors as stimuli for the experimental design (Figures 1a–c). Thus, the sensory stimuli we used in the laboratory experiment were part of the company’s corporate identity. Each stimulus was evaluated by 20 subjects. During the testing process, the participants in the experiment were not informed that the stimuli were presented by the Cartier Dom, therefore, we avoided the brand’s influence on perception.

#### 2.1.3 Design and procedure

**Figure 2:**
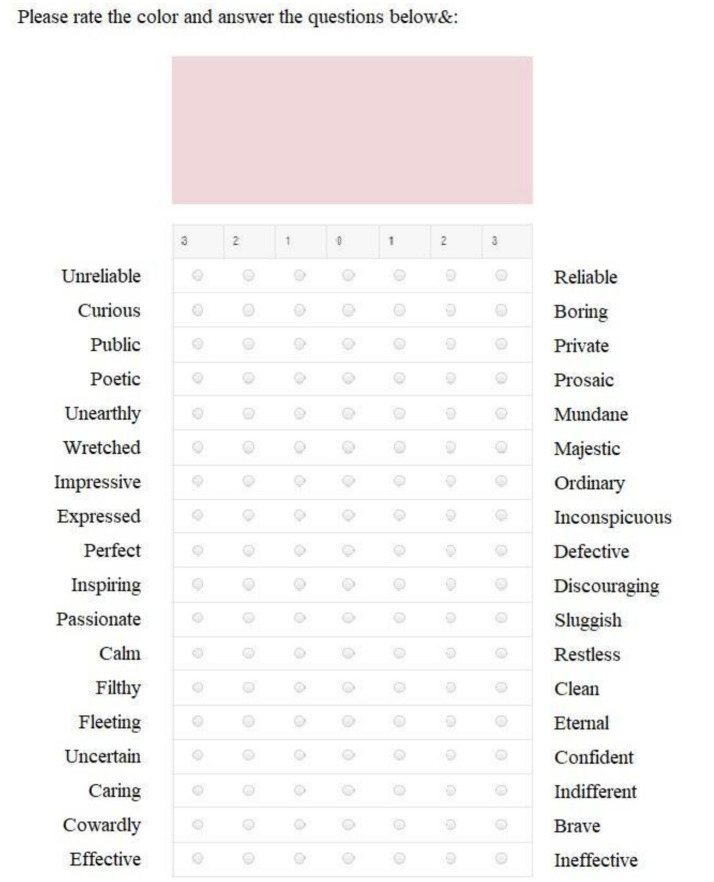
Assessment of sensory stimuli based on the C. Osgood SD questionnaire.

**Figure 3:**
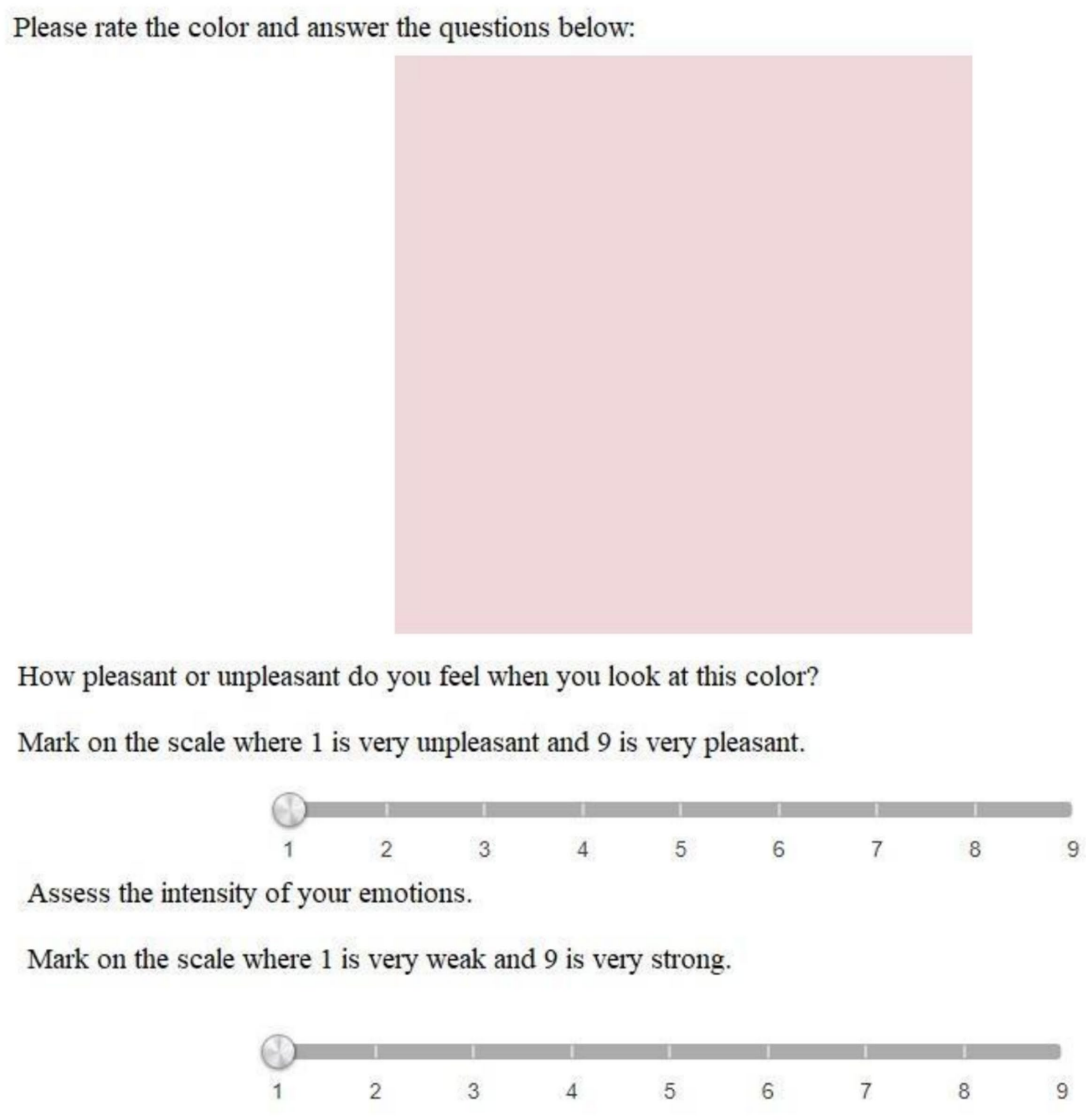
Assessment of sensory stimuli by valence/arousal.

We applied SD (Osgood, 1964) to characterize the target audience’s emotional attitudes toward the colors, odor, and music associated with the retail zones. Trial tests have shown that it is more comfortable for participant to evaluate half of the stimuli (23/24). Therefore, each participant tested half of the sensory stimuli on SD scale (Figure 2). We also assessed the valence and arousal of the emotions evoked by the stimuli to avoid those with a high negative valence. Following Lang et al. (1999), valence was assessed by our participants from 1 (very negative) to 9 (very positive), whereas arousal could range from 1 (very weak) to 9 (very strong; Figure 3). These 9-unit scales are very common both in affective psychophysiology (Golonka et al., 2017) and general psychology (Kosonogov et al., 2020). These scales, introduced by Lang et al. (1999), allow participants to evaluate valence from 1 (very negative) to 9 (very positive) and arousal from 1 (very weak) to 9 (very strong; Figure 3). The wording was as follows: unpleasant/pleasant, low/high arousal.

The order of stimulus presentation was randomized. The test duration was about 50 min for each participant.

To analyse the SD data, we computed the mean scores of all respondents for each sensory stimulus. This allowed us to generate heatmaps depicting the associative strength between the stimuli and the adjectives. In a similar vein, valence and arousal ratings were evaluated for each sensory stimulus. To assess the consistency among the adjectival descriptions for each retail zone (e.g., calm, private, caring, confident, effective, and reliable for the checkout area) and identify potential latent contradictions, we conducted a factor analysis. IBM SPSS Statistics 27 was used for all statistical computations. The questionnaire was delivered to the participants using the EnjoySurvey resource^1^ (Figures 1, 2).

### 2.2 Phase 2: neurophysiological assessment of emotional reactions to the multisensory retail stimulation

The second stage involved measuring EDA, HR, and HRV to obtain objective information about the emotional and physiological reactions of consumers in the modulated multisensory environment and to determine whether they characterize approach or avoidance behavior. This stage also involved the creation of relevant multisensory spaces by using the stimuli from the first stage as well as immersive technology to demonstrate color in the virtual rooms, headphones to present music, and odor samples.

#### 2.2.1 Participants

Twenty-seven subjects (33.6% men, 67.4% women; age = 23–35 years) provided their informed consent to participate in the experiment. All procedures were conducted in accordance with the Helsinki Declaration and approved by the ethics committee of the Higher School of Economics. The subjects received compensation from the company for their participation.

#### 2.2.2 Stimuli

Nine multisensory virtual rooms, each rendered in a distinct color corresponding to our experimental conditions, were developed in the second stage of the study. The «Unity platform» was used to develop these virtual environments. Participants wore an «HTC Vive» headset and were positioned at the center of each virtual room. Simultaneously, musical pieces were played through headphones. The most appropriate visual, auditory, and olfactory stimuli were selected based on the results obtained in the first stage (Tables 1–3, see Tables 4, 5 for the most relevant sensory stimuli for the retail zones). The intensity of the red color saturation corresponded positively with the relevance of the sensory stimuli to the associated adjectives. The sensory stimuli we used in the study were characterized by positive or neutral valence, with no negative valence stimuli included. Consequently, valence was not considered as a selection criterion in the stimulus set. The observed patterns in valence and arousal may be attributed to the proprietary incentives supplied by the company, which were designed to elicit exclusively positive responses from the target audience. The subjects were in each environment for 1 min. Then, similarly to the first stage, the investigator asked them to assess valence of the experience on the scale from 1 (very negative) to 9 (very positive) and arousal from 1 (very weak) to 9 [very strong; as in Lang et al. (1999)].

**Table 1:** The results of the valence and arousal assessment of the relevance of Visual stimuli (color, texture) to individual adjectives or a group of adjectives describing shopping space zones (Heat map: red saturation shows the most suitable stimuli, blue saturation shows unsuitable ones).

Stimulus*	Stimulus valence	Stimulus arousal	Welcome	Cashier	Wedding rings	Diamonds
C1	0.21	−1.74	−1.70	1.80	0.09	−1.06
C10	0.55	−1.05	−0.85	1.80	0.25	−1.00
C11	0.75	−0.45	−0.80	1.00	0.59	−0.58
C12	−0.45	0.85	1.00	−1.80	−0.61	1.36
C13	−0.4	−0.55	−0.75	0.15	−1.24	−0.51
C14	0.35	−0.05	−0.40	−0.10	−0.73	0.11
C2	0.26	−1.74	−0.75	1.05	−0.76	−0.48
C3	0.11	−1.89	−0.80	1.55	0.45	−0.65
C4	−0.37	−1.89	−1.60	0.65	−0.35	−0.94
C5	0.68	−0.89	−0.35	1.70	0.00	−0.18
C6	1.28	−0.22	0.60	1.35	0.73	0.08
C7	1.67	−0.22	1.20	0.60	0.28	0.60
C8	1.45	−0.05	−0.10	1.55	0.54	0.04
C9	1.15	−0.40	−0.15	1.50	0.74	−0.36
T1	−0.79	−1.26	−0.65	0.50	−0.84	−0.85
T12	−0.16	0.26	1.10	−1.30	−0.39	0.71
T13	−1.15	−0.25	−0.10	−1.25	−1.36	0.04
T14	−0.35	0.55	0.15	−1.15	−0.61	0.53
T2	−0.22	0.22	−1.00	−0.70	−1.08	−0.69
			Zones			

*С1–С9, Т1, Т2, Т12, Т13, Т14: encoding of color (see Figure 1).

**Table 2:** The results of the assessment of valence and arousal of olfactory stimuli (fragrances) to individual adjectives or a group of adjectives describing shopping space areas (Heat map: red saturation shows the most suitable stimuli, blue saturation shows unsuitable ones).

Stimulus*	Stimulus valence	Stimulus arousal	Welcome	Cashier	Wedding rings	ZONE: jewelry made of precious stones
P1	−0.11	0.95	0.35	−1.20	−0.20	0.74
P2	0.74	0.84	1.55	−0.85	0.41	1.11
P3	1.11	−0.22	1.10	0.05	0.31	1.19
P4	0.40	0.75	0.70	−0.40	−0.06	0.93
P5	0.50	1.15	0.70	−0.15	−0.11	1.46
P6	−0.85	0.05	0.40	−0.75	−0.64	0.34
P7	−0.70	0.50	−0.15	−1.15	−0.80	0.49

*P1–P7: encoding of olfactory stimuli (see Figure 1).

**Table 3:** The results of the valence and arousal of auditory stimuli (music) to individual adjectives or a group of adjectives describing retail space zone (Heat map: red saturation shows the most suitable stimuli, blue saturation shows unsuitable ones).

Stimulus*	Stimulus valence	Stimulus arousal	ZONE: welcome	ZONE: cashier	ZONE: wedding rings	ZONE: jewelry made of precious stones
S1	2.26	0.89	0.80	1.70	1.60	0.59
S10	1.83	0.72	1.15	−1.45	1.73	1.15
S11	1.85	0.60	1.20	0.30	0.44	0.25
S12	0.55	0.35	0.60	−0.35	0.45	−0.21
S13	1.10	−0.25	0.00	0.35	−0.41	0.36
S14	1.20	0.95	0.60	1.80	0.83	0.80
S15	1.10	0.25	0.90	0.05	0.29	0.69
S16	1.55	0.60	1.05	0.80	0.35	0.89
S17	2.00	1.25	1.85	−0.05	0.44	1.20
S18	−0.35	0.10	−0.35	0.75	−0.49	−0.59
S19	1.05	−0.15	0.15	0.90	0.76	0.28
S2	1.79	1.00	1.40	−0.60	0.11	1.33
S20	0.60	0.85	1.10	0.80	0.94	1.25
S21	0.70	0.00	0.85	0.00	−0.13	0.66
S3	2.32	0.05	0.30	2.05	1.28	0.13
S4	2.05	0.26	1.35	0.85	0.63	0.76
S5	0.68	−0.42	0.35	0.35	0.23	0.31
S6	−0.17	−0.56	0.45	−0.30	0.11	0.09
S7	1.50	0.39	1.16	0.00	0.34	0.86
S8	0.28	−0.44	0.50	0.20	0.46	0.41
S9	0.00	−0.89	−0.37	−0.11	−0.54	−0.04

*S1–S21: encoding of auditory sensory stimuli (see Figure 1).

**Table 4:** The most relevant sensory stimuli to a single adjective or group of adjectives describing areas of the retail space.

Stimuli/Zone	Welcome	Cashier	Wedding rings	Diamonds
Visual	C7, C12, T12	C1, C5, C10,	C6, C9, C11	С7, C12, T12
Olfactory	P2, P4, P5	P3	P2, P3	P3, P2, P5
Auditory	S2, S4, S17	S1, S3, S14,	S3, S10,	S2, S10, S17

C, T, P, S Encoding of sensory stimuli (see Figure 1).

**Table 5:** Encoding sensory stimuli to create multisensory environments.

	ZONE: welcome	ZONE: cashier	ZONE: wedding rings	ZONE: jewelry made of precious stones
Multisensory rooms (1)–(9)	(1) C7, P2, S2	(2) C1, P3, S1	(3) C6, P2, S3	(4) С7, P3, S2
	(5) C12, P4, S4	(6) C5, P3, S3	(7) C9, P3, S10	(8) T12, P5, S17
	(9) T12, P5, S17			

C, T, P, S Encoding of sensory stimuli (see Figure 1).

Olfactory stimuli were presented using a cup with a closable lid. The experiment was conducted in a laboratory setting, where the participant was accompanied by an experimenter who assisted with the presentation of odors. At the beginning of each room demonstration, the experimenter selected the appropriate odor sample, opened the lid, and held it approximately 5–7 cm from the participant’s nose.

The selection of stimuli was derived from the quantitative results of the first stage of the study (Tables 1–5). Using the semantic differential method, we identified stimuli that showed strong and exclusive associations with individual descriptive adjectives while minimizing overlap with others, thereby ensuring semantic coherence and enabling the detection of latent contradictions. Final multisensory combinations of color, scent, and music were determined through cross-analysis of Tables 4, 5, prioritizing triplets that were statistically associated with the same adjective to produce a coherent multisensory impression. The experiment was limited to nine rooms to balance ecological validity with participant comfort and to reduce the risk of fatigue.

#### 2.2.3 Physiological recording and processing

Physiological signals were collected, amplified, and filtered using the ActiChamp data acquisition system (Brain Products, Germany). The recording was conducted at a sampling frequency of 500 Hz. Bipolar electrodes were placed on the index and ring fingers of the left hand of each participant to obtain skin conductance signals, while a photoplethysmograph was positioned on the middle finger to measure heart rate. To process peripheral nervous system data, we used the Lab Chart Reader software. Prior to analysis, each file was visually inspected and cleared of artifacts. Raw EDA signals were calibrated to capture activity within the 0–100 μS range. To correct for distorted data distribution, a transformation was applied using the formula log10 (EDA + 1). We marked all peaks during a period of 1 min (the duration of demonstrating a single virtual room), calculated the difference between the maximum and minimum values, and then summed this difference. Heart rate, which was expressed in beats per minute (bpm), was determined based on peak amplitude. We then calculated the SDNN (standard deviation of time between successive heartbeats) to estimate HRV. Outliers (values 3 SD greater than the mean) were excluded.

We chose SDNN as our primary HRV index because it offers a reliable measure of overall autonomic nervous system variability and is well-suited for short-term recordings, such as the 1-min intervals used in our study. Unlike RMSSD or pNN50, which primarily reflect parasympathetic tone, SDNN captures both sympathetic and parasympathetic contributions to HRV (Shaffer and Ginsberg, 2017; Laborde et al., 2017). It has also been widely used in affective neuroscience and psychophysiological research to track changes in emotional valence and regulatory flexibility (Kreibig, 2010; Kim et al., 2018). Furthermore, SDNN is less susceptible to transient artifacts and variability caused by movement (Besson et al., 2025), which is especially relevant in immersive VR environments. While frequency-domain indices such as LF/HF can provide additional insights, they typically require longer continuous recordings (2–5 min) and were thus not feasible given our experimental design.

#### 2.2.4 Procedure and analysis

**Figure 4:**
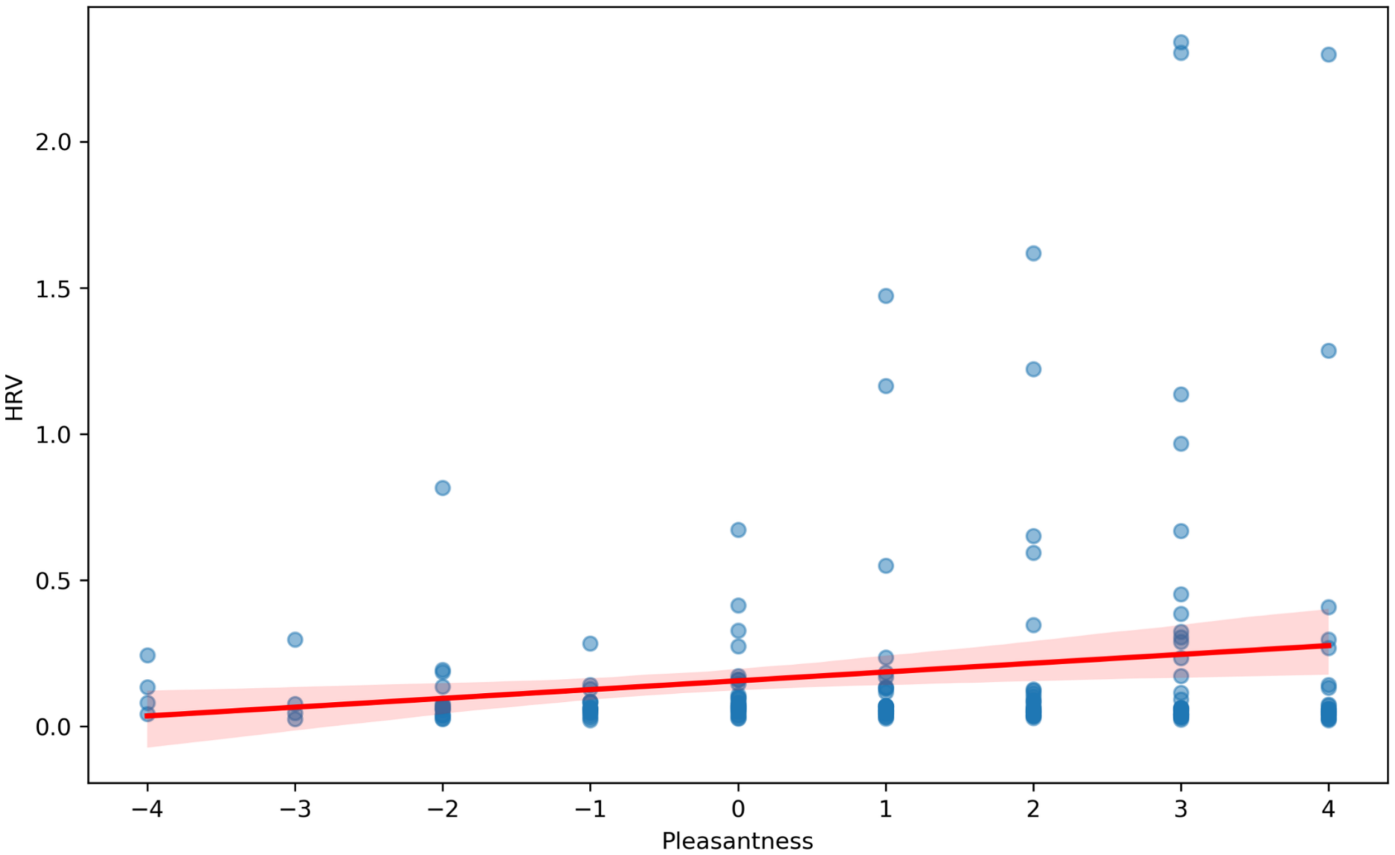
Correlation analysis with repeated measurements between HRV (seconds) and hedonistic assessments (from −4 to +4) of virtual rooms by study participants.

**Figure 5:**
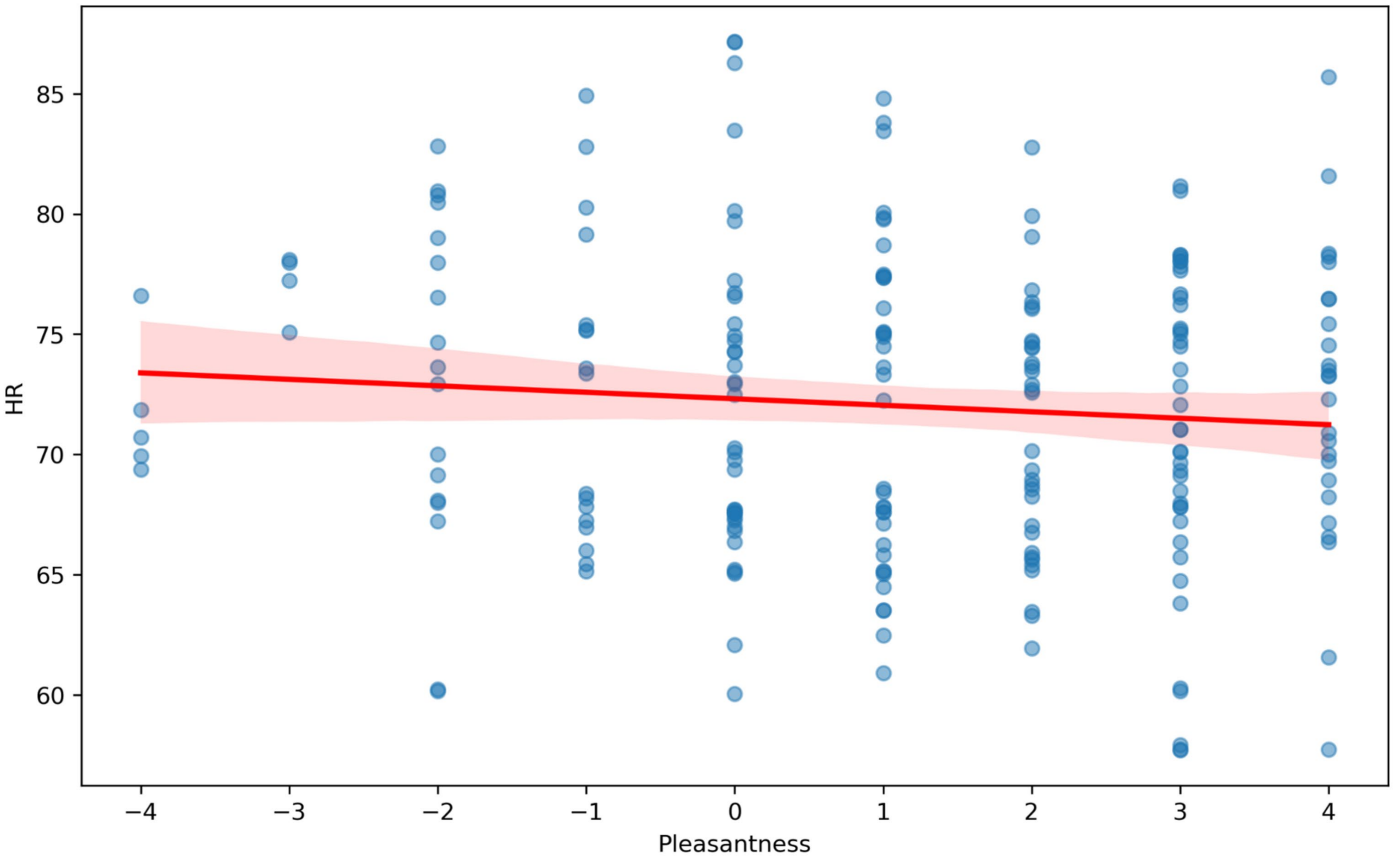
Correlation analysis with repeated measurements between HR (beats per minute) and hedonistic assessment (Pleasantness) (from −4 to +4) of virtual rooms by study participants.

**Figure 6:**
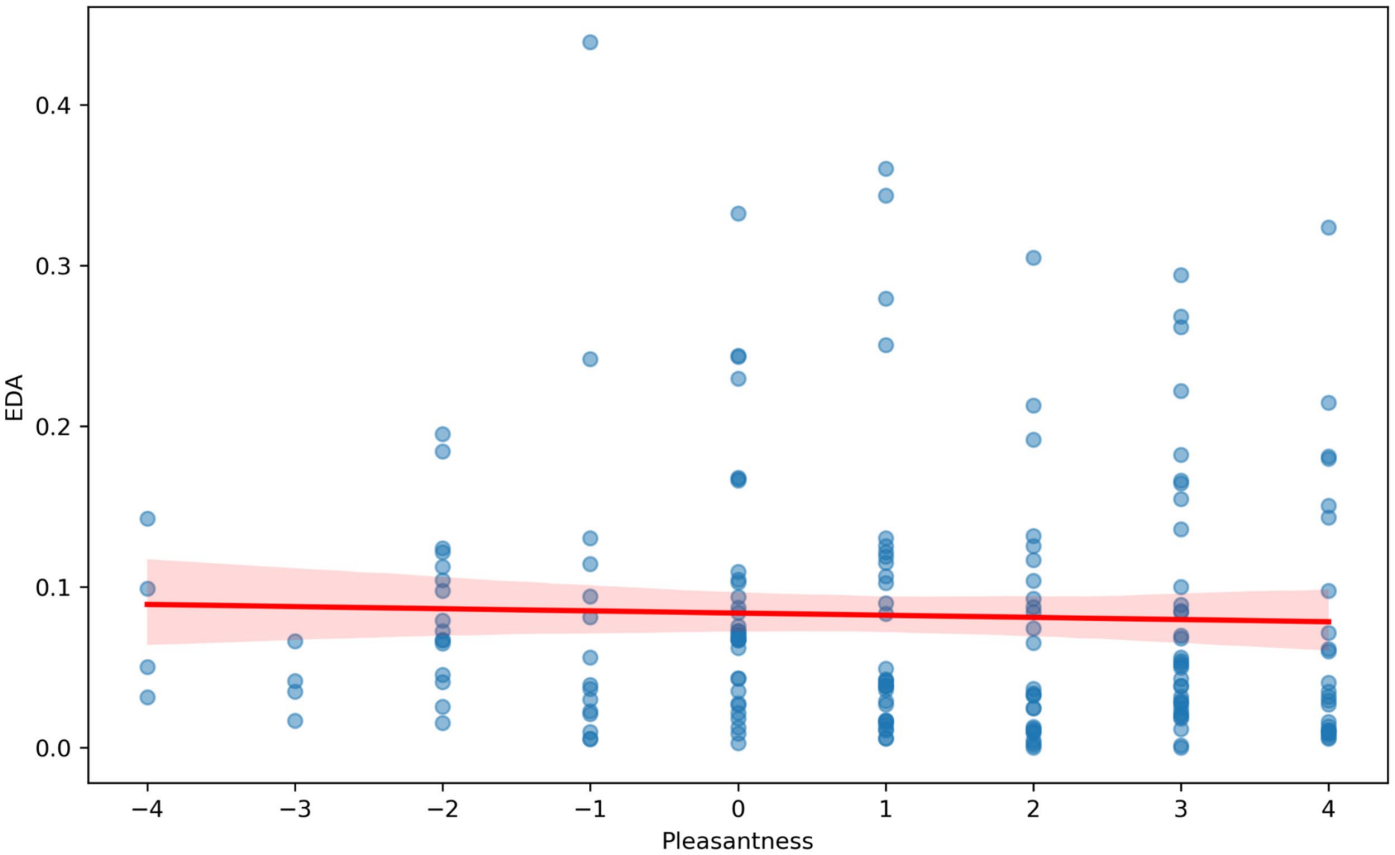
Correlation analysis with repeated measurements between EDA (μS) and hedonistic assessments (Pleasantness) (from −4 to +4) of virtual rooms by study participants.

The VR immersion of participants in the multisensory environments took place in an experimental laboratory. A VR display with built-in headphones was used to demonstrate the colors and sounds in the virtual reality rooms. The odors were presented in special containers, with the lid at a distance of 7 cm from the subject’s nose. The subjects were in each modulated environment for 1 min. Then, similarly to the first stage, the investigator asked them to assess valence from 1 (very negative) to 9 (very positive) and arousal from 1 (very weak) to 9 [very strong; as in Lang et al. (1999)]. Rmcorr was used to calculate the correlation with repeated measures (within-individual linear associations for paired data) (Bakdash and Marusich, 2017; Marusich and Bakdash, 2021) (Figures 4–6). For the repeated-measures correlation (rmcorr) analysis, the independent variable was the hedonic evaluation of virtual rooms on a − 3 to +3 Likert scale, and the dependent variables were VHR, HR, and EDA. Participant ID served as the repeated-measures factor, accounting for the non-independence of multiple observations within individuals. This method was chosen because the data consisted of paired observations (evaluation and physiology) across several rooms per participant. The analysis revealed a positive within-subject association (r_rm = 0.17, *p* = 0.02), indicating that more favorable evaluations of a room were linked to higher heart rate variability.

## 3 Results

### 3.1 Phase 1

Based on the SD data analysis, we identified associations between the sensory stimuli and the adjectives characterizing the distinct retail zones. Mean rating scores derived from the SD scale were calculated for each sensory stimulus and subsequently subjected to factor analysis (Table 6). The analysis revealed inconsistencies arising from the intuitive, adjective-based approach used to characterize the retail zones. For example, the checkout area was described using the adjectives “calm” and “efficient,” which was a contradictory pairing. The Diamond and Wedding Rings zones had the most consistent and coherent descriptions. Based on the results of the factor analysis (Table 6), we modified the adjectives used to position the various zones within the retail space (Table 7). The Welcome zone proved to be the most contentious; ultimately, we decided to retain the adjective “curious,” as it best represented the company’s brand concept and did not elicit contradictory responses from the target audience.

**Table 6:** Results of the PCA analysis of adjectives characterizing shopping zones.

Zone/Variables		High arousal sensory stimuli	High valency sensory stimuli	Low arousal sensory stimuli
Welcome	Inspiring	0.63	0.66*	0.25
	Curious	0.85*	0.40	−0.18
	Majestic	0.51	0.68*	0.34
Cashier	Calm	−0.49	0.42	0.58*
	Private	−0.37	0.75*	0.13
	Caring	0.30	0.69*	0.19
	Confident	0.93*	−0.07	0.16
	Effective	0.82*	0.15	0.38
	Reliable	0.08	0.11	0.90*
Wedding rings	Perfect	0.22	0.67*	0.61*
	Pure	0.00	0.77*	0.42
	Eternal	−0.36	0.03	0.47
	Ethereal	0.05	0.91*	−0.05
	Poetic	0.21	0.91*	−0.05
Diamonds	Passionate	0.92*	0.18	−0.15
	Bold	0.92*	0.02	−0.07
	Expressive	0.83*	0.05	−0.36
	Impressive	0.71*	0.57	−0.16

**Table 7:** Semantic associations of the retail space zones based on the PCA results.

Retail space areas	Starting semantic associations of shopping space areas	Adjusted semantic associations of shopping space areas
Welcome	Inspiring	Curious
	Curious	
	Majestic	
Cashier	Calm	Calm
	Private	
	Caring	
	Confident	
	Effective	
	Reliable	
Wedding rings	Perfect	Perfect
	Pure	Pure
	Eternal	Ethereal
	Ethereal	Poetic
	Poetic	
Diamonds	Passionate	Passionate
	Bold	Bold
	Expressive	Expressive
	Impressive	Impressive

The obtained adjective scores were summarized and averaged as global rating data for each area, taking into account the changes resulting from the factor analysis (Table 7). A factor analysis was conducted on the 18 adjectival descriptors to examine their underlying structure and assess consistency across retail zones. Principal Component Analysis (PCA) with Varimax rotation (three factors specified *a priori*) yielded a stable solution explaining 80.18% of the total variance. The rotated structure revealed three coherent factors: high-arousal stimuli (e.g.*, Curious, Bold, Expressive*), positively valenced stimuli (e.g., *Pure, Ethereal, Caring*), and low-arousal stimuli (e.g., *Reliable, Calm*). Most descriptors exhibited high communalities (>0.75), confirming the adequacy of the solution, while a few (e.g., *Majestic*) showed weaker fit. This factor structure provided the basis for evaluating the consistency and potential contradictions in adjectival associations across zones.

The maximum and minimum scores obtained upon assessing the relevance of the sensory stimuli in the selected areas were +3 and −3, respectively. Thus, the highest rates for the evaluated odors and music best corresponded to the following shopping zones: Welcome, Cashier, Wedding Rings, and Diamond (Tables 1–3). In addition, the data analysis revealed a highly positive Pearson correlation (*r* = 0.798, *p* = 0.001) between the valence and SD scores for the Wedding Rings zone (*r* = 0.809, *p* < 0.001) and (*r* = 0.769, *p* = 0.001) between the arousal and SD scores for the Diamond and Welcome Zones. These results indicate that sensory stimuli with cross-modal correspondences with the adjectives “perfect,” “pure,” “ethereal,” and “poetic” had higher positive valences, whereas stimuli corresponding to the adjectives “curious,” “passionate,” “bold,” “expressive,” and “impressive” were perceived as more intense. A negative correlation (*r* = −0.441, *p* = 0.002) was observed between the arousal scores and SD scores the for the Cashier zone, suggesting the need for sensory stimuli with low levels of emotional arousal in this area.

The mean valence and arousal ratings for the sensory stimuli indicated that all stimuli presented by the company were perceived as pleasant and neutral. This verification was necessary to exclude any aversive stimuli from further consideration.

As a result of conducting factor analysis, we were able to refine the adjectives describing four zones of the retail space: Welcome (1)—Curious; Cashier (2)—Calm; Wedding Rings (3)—Perfect, Pure, Ethereal, Poetic; Diamonds (4)—Passionate, Bold, Expressive, Impressive. The adequacy of the adjective structure was assessed through evaluation of internal consistency using Cronbach’s alpha coefficient.

Factors (1) and (2) demonstrated satisfactory internal consistency (*α* = 0.634 and *α* = 0.633, respectively). These four factors correspond to different areas within the retail space: ZONE: welcome; ZONE: cashier; ZONE: wedding rings; ZONE: jewelry made of precious stones. Although these values are slightly below the conventional cutoff of 0.7, they are considered acceptable within psychometric and sociological research contexts, particularly given the large number of items (*N* = 47), indicating sufficient reliability of these subscales. Factor (4) exhibited good internal consistency (*α* = 0.771), reflecting high reliability and homogeneity of this subscale. This value surpasses the commonly accepted threshold of 0.7, allowing confident use of this factor in subsequent analyses. Factor (3) showed low reliability (*α* = 0.500), indicating inadequate internal consistency. This result suggests that the items within this factor may measure heterogeneous constructs or require further modification and refinement. Therefore, the interpretation of results related to Factor 3 should be approached with caution. In summary, three of the four identified factors (1, 2, and 4) demonstrated acceptable to good levels of reliability. The low reliability of Factor 3 represents a limitation of this study and warrants further consideration.

Lance et al. (2006) provided a detailed analysis of the origins of widely used threshold values (e.g., 0.70, 0.90) in methodological research. They found that these thresholds were selected arbitrarily and are often misinterpreted in contemporary contexts. The authors emphasize the need for a more flexible and contextual approach to the use of such criteria, rather than blind adherence to established figures. Hinkin (1998) explicitly states that the reported reliability coefficient (e.g., an internal consistency measure such as Cronbach’s alpha) should be equal to or exceed 0.60 during the development and validation stages of new scales. Specifically, on page 15, the author emphasizes that a reliability level of approximately 0.60 or higher is generally considered acceptable for new or experimental scales to ensure a minimal degree of consistency among scale items. This serves as a standard benchmark in measurement development practice, especially in the early testing phases, while more stringent criteria (e.g., 0.70 or higher) are recommended for final versions of the scales.

### 3.2 Phase 2

We conducted a correlation analysis with repeated measures to determine the relationship between HR, HRV, and EDA and the valence ratings of virtual rooms with various sensory stimuli. The individual results showed that HRV was significantly increased in pleasant rooms (Figure 4) [rrm (185) = 0.15, 95% CI (0.006, 0.286), *p* = 0.042]. Our results also revealed a trend toward a decrease in EDA and HR in pleasant room conditions. The correlation between HR and valence ratings (Figure 5) was not significant [rrm (191) = −0.09, 95% CI (−0.232, 0.048), *p* = 0.196]. The correlation between EDA and valence was not significant (Figure 6) [rrm (191) = −0.08, 95% CI (−0.223, 0.077), *p* = 0.332].

Using G*Power 3.1, we conducted sensitivity analyses assuming a within-subject repeated measures correlation design (as in our main analyses using rmcorr). For the significant correlation between HRV and valence (rrm = 0.15), the analysis yielded a power of 0.64 (*α* = 0.05, *n* = 27, 186 observations). In contrast, the non-significant correlations for HR (rrm = −0.09, 192 observations) and EDA (rrm = −0.08, 192 observations) showed lower statistical power: 0.30 and 0.26, respectively. These results suggest that while the study had moderate power to detect small-to-medium effects in HRV, it was underpowered to reliably detect small effects in HR and EDA. Future studies should consider increasing the sample size or extending the duration of exposure to improve the detection of subtle autonomic responses in multisensory retail environments.

## 4 Discussion

Our study was conducted to model and objectively test the specific emotional experiences of consumers in various areas of a retail space. We specifically aimed to assess their approach or avoidance behaviors based on multisensory stimuli and electrophysiological measurements of their peripheral neural responses.

We used the SD test results to select the most suitable sensory stimuli for the different areas of the retail space. This allowed us to adjust the combinations of sensory stimuli (color + texture, music, and odor) that differentiated the specific zones (Welcome, Cashier, Wedding Rings, Diamond) and determine their correspondence with their semantic descriptions. The SD results showed that the descriptions of the zones sometimes overlapped, leading to unclear positioning and perception among target consumers. The SD results can be considered the basis for informatively replacing the intuitive expert approach with a scientific one. Based on the SD test, we not only adjusted the initial list of adjectives describing the positioning of the various retail areas but also obtained the most suitable sensory stimuli for them. We then used these stimuli to create multisensory environments in VR and tested them using neurophysiological methods.

Researchers in sensory marketing have previously discussed the benefits of multisensory experiences in organizing retail spaces. For example, Duong et al. (2022) showed that an increase in the number of sensory modalities stimulating consumers’ emotional reactions resulted in the formation of positive attitudes toward the brand. Spangenberg et al. (2005) showed that multisensory environments can enhance consumers’ emotional experiences in retail environments. However, deciding upon an appropriate combination of sensory stimuli is a difficult, sometimes controversial task. Our multisensory stimuli involved combinations of 14 colors, 5 textures, 21 music pieces, and 7 odors that best matched each of the four retail space zones: Welcome, Cashier, Wedding Rings, Diamond. To identify the most effective combination of sensory stimuli corresponding to positioning, we considered the association between emotional valence and SD score and found that the Diamond and Welcome zones had high positive associations in this regard. This indicates that sensory stimuli with cross-modal correspondences with the adjectives “perfect,” “pure,” “ethereal,” and “poetic” had higher positive valences, whereas stimuli corresponding to the adjectives “curious,” “passionate,” “bold,” “expressive,” and “impressive” were perceived as more arousing.

Studies have demonstrated that odors can modulate autonomic responses and emotional states, often without conscious awareness, which is particularly relevant in retail environments. For instance, Sayorwan et al. (2012) showed that lavender scent reduced sympathetic activity and increased parasympathetic activity, which was reflected in HRV changes. Similarly, Sowndhararajan et al. (2015) used EEG and physiological signals to show reduced arousal and increased relaxation in response to essential oils. Guéguen and Petr (2006) demonstrated that ambient odors (such as citrus and cleaning scents) could significantly influence consumer dwell time and purchasing behaviors through unconscious mechanisms. De Jesus Junior et al. (2023) showed that the addition of natural scents to VR environments improved relaxation and HRV metrics, while Song et al. (2024) demonstrated similar effects in urban green space simulations. Furthermore, fNIRS-based studies (Zhou et al., 2023; Molina-Rodríguez et al., 2023) have confirmed the sensitivity of prefrontal activity to scent-induced emotional changes, even in imagined scenarios. These studies align with our findings and provide independent support for the reliability of physiological metrics in capturing olfactory-induced emotional changes.

Based on principal component analysis (PCA) of the SD scores, we identified new associations for the shopping zones and tested them in the neurophysiological part of the experiment. This approach resonates with kansei engineering (Nagamachi, 1994), which was conceived for industrial design in the 1960s to evoke specific emotional responses in customers through the design of products and services. Kansei engineering parametrically links consumer emotions and product features, allowing for predictions of aggregated choices in the market. We complemented the SD-based kansei approach with the use of neurophysiological methods, such as EDA, HR, and HRV measurements.

We showed that positively ranked multisensory retail environments were associated with increased HRV. Further, we observed only a trend toward a decrease in EDA and HR in pleasant room conditions. Our results are in line with those of previous studies which have shown that HRV usually increases in pleasant conditions (Kumpulainen et al., 2024; Benz et al., 2022). However, contrary to the previous results, neither EDA nor HR was statistically significantly associated with emotional valence, which indirectly indicates their low sensitivity to multisensory stimuli when compared to HRV, probably due to high intersubject variability. Although the trend toward a reversed HRV association between valence and EDA and valence and HR can be indirectly supported by the literature results (Yu et al., 2024), a firm conclusion cannot be made regarding the HR and EDA markers of hedonic assessment in multisensory environments based on our results, which is a limitation of our study.

Our findings align with the literature on the restorative effects of multisensory experiences on physiological states. Song et al. (2024) highlighted that a multisensory stimulation in a virtual urban green space environment can lead to significant physiological recovery, as evidenced by decreased blood pressure and improved overall health metrics among participants exposed to various sensory stimuli. The positive correlation between HRV and multisensory environments indicates that such settings may foster a relaxed and adaptive physiological state, potentially mediated by the vagus nerve’s influence on heart function.

However, our observations of a trend toward decreased EDA and HR under pleasant room conditions warrant further discussion. While one might expect a direct correlation between pleasant environments and reduced stress indicators such as EDA and HR, our results suggest a more nuanced relationship. Previous studies have shown that pleasant environments can enhance emotional wellbeing but do not always correlate with immediate physiological changes in EDA and HR (Spence et al., 2014). This complexity may arise from individual differences in sensory processing or the specific nature of the stimuli presented. Additionally, the interplay between various sensory modalities may contribute to this phenomenon. It is possible that pleasant conditions made the participants feel positive, but the physiological indicators did not reflect an immediate reduction in arousal due to the situational context or cognitive appraisals of the environment. HR and EDA responses are known to exhibit substantial between-subject variability, influenced by stable traits (e.g., individual differences in autonomic reactivity, personality, or baseline arousal levels) as well as temporary states (e.g., fatigue, mood, stress). As Kreibig (2010) emphasizes, emotional modulation of HR and EDA may be weak or inconsistent at the group level if individual baseline differences are not accounted for or if variability masks subtle stimulus effects. In immersive VR settings, participants are often subject to movement-related artifacts and attentional fluctuations, both of which can introduce noise into autonomic signals. Although we visually inspected and cleaned the data, residual motion or engagement-related variance could have impacted the EDA and HR signal integrity more than HRV. Unlike HRV, which is computed over a window of interbeat intervals and is less sensitive to individual peaks or outliers, raw HR and EDA values can be disproportionately influenced by transient changes in respiration, posture, or skin contact with electrodes. HRV (especially SDNN and RMSSD) reflects complex interactions between sympathetic and parasympathetic branches of the ANS and is often more sensitive to subtle changes in emotional valence compared to HR or EDA, which are predominantly arousal-linked.

We used immersive VR environments in the second stage of the study. Many studies have shown that VR environments can effectively simulate reality and are suitable for hypothesis testing and scientific research. For example, Armougum et al. (2019) showed that the markers of cognitive workload during orientation tasks did not differ between real performance at a train station and the virtual environment simulating it. Using VR, we immersed our participants in multisensory environments involving all three modalities—visual, auditory, and olfactory—and measured the participants’ physiological reactions. Specifically, we assessed their self-reported (valence and arousal) and physiological (HR, HRV, and EDA) markers of emotion in response to the stimuli. Our findings align with those of prior studies involving immersive VR environments that have highlighted the role of multisensory stimuli in enhancing physiological and psychological states. For instance, De Jesus Junior et al. (2023) demonstrated that sensory elements such as natural sounds and smells improve relaxation and reduce stress, as measured by HRV metrics. These results suggest that the integration of multiple sensory modalities can enhance people’s sense of presence and immersion, in turn fostering physiological recovery and emotional balance.

Our findings offer several practical applications for businesses seeking to enhance retail environments and brand strategy through sensory design. First, the use of SD ratings and physiological data enables the zoning of retail spaces based on specific emotional profiles. For instance, environments that aim to evoke feelings of purity and romance (e.g., wedding ring displays) should include sensory stimuli with low arousal and high positive valence—such as soft lighting, floral scents, and slow, melodic music. In contrast, zones intended to elicit excitement and boldness (e.g., diamond displays) benefit from high-arousal stimuli, including saturated colors, rich scents, and stimulating soundtracks. Second, brands can use the combined SD and biometric methodology presented here to pre-test their aesthetic and atmospheric elements—such as store playlists, packaging visuals, or signature scents—before deploying them across retail formats. This supports data-driven sensory branding strategies that move beyond intuition or anecdotal feedback. Third, the observed association between positive emotional valence and increased HRV suggests that emotionally congruent multisensory environments promote a physiological state conducive to customer engagement, comfort, and potentially prolonged dwell time. Retailers can strategically apply this insight to high-value zones, service counters, or areas where increased interaction is desirable. Finally, the use of noninvasive physiological monitoring tools (e.g., wearable HR sensors) opens the door to integrating biometric feedback into smart retail systems. Businesses could use these insights to A/B test different store layouts or even create adaptive retail environments that adjust lighting, scent, or sound in real time based on customer traffic patterns or biometric trends.

From a managerial perspective, our results indicate that HRV may be used as a primary physiological key performance indicator for pre-testing store atmospherics, as it reflects vagal tone and adaptive self-regulation and is consistently associated with restorative, low-stress states (Shaffer and Ginsberg, 2017; Laborde et al., 2017). The study further underscores the importance of designing environments based on multisensory congruence. Prior work has shown that alignment across sensory modalities, such as music and scent, improves store evaluations and approach behavior, while incongruence can attenuate these effects (Spangenberg et al., 2005; Spence, 2020). In addition, the present results suggest that the calibration of scent intensity plays a critical role in shaping consumer responses. Evidence from retail contexts indicates that moderate and contextually congruent scents enhance mood and evaluation, whereas overly intense scents may have counterproductive effects, particularly under conditions of time pressure (Leenders et al., 2019). These findings also highlight the value of integrating semantic differential measures with biometric indices such as HRV to inform the design of specific retail zones. Virtual reality may serve as a useful pre-test environment, given its capacity to reliably evoke affect and presence, thereby enabling agile testing of store concepts prior to costly in-store implementation (Assiouras et al., 2024). Nonetheless, the ultimate effectiveness of these design strategies requires field validation, where physiological indices should be linked to behavioral outcomes such as dwell time, purchase volume, and repeat visits.

### 4.1 Limitations and directions for future research

Several limitations of the study should be acknowledged. The relatively short exposure duration of approximately 1 min constrained the HRV analyses, as frequency-domain measures typically require at least 2 to 5 min of continuous data (Shaffer and Ginsberg, 2017; Laborde et al., 2017). Future research should therefore employ longer exposures or repeated measurement blocks to allow for both time- and frequency-domain HRV analyses, thereby increasing sensitivity. Moreover, although HR and electrodermal activity demonstrated general trends, their lack of significant valence effects is consistent with the literature that emphasizes considerable interindividual variability in autonomic responses (Kreibig, 2010). Subsequent studies should therefore consider larger sample sizes, advanced mixed-effects modeling, and the inclusion of additional physiological controls such as respiration or skin temperature.

As shown in the Results section, the Cronbach’s alpha values for two retail space zones (Welcome 1 and Cashier 2) demonstrated satisfactory internal consistency (*α* = 0.634 and *α* = 0.633, respectively). Although these values are slightly below the conventional threshold of 0.70, they are considered acceptable in research practice (Lance et al., 2006; Hinkin, 1998). A more notable limitation is the low internal consistency of the adjectives for Zone 3 (Wedding ring) (*α* = 0.5), which calls into question the reliability of the measurements for this specific construct and necessitates a highly cautious interpretation of the corresponding results. Future research is recommended to revise and validate this subscale.

The olfactory stimuli in the current study were delivered using open containers, which limited control over concentration and temporal dynamics and may have introduced adaptation effects. Prior research has documented that olfactory habituation can substantially attenuate perceptual and physiological responses with repeated exposure (Dalton, 2000). The use of computer-controlled olfactometers and the careful calibration of scent intensity would allow for more precise experimental manipulation in future studies. Another limitation lies in the restricted valence range of the stimuli, which were predominantly neutral-to-positive. This limited variability may have constrained the detection of effects for measures such as HR and electrodermal activity. Future research should incorporate stimuli spanning the full valence-arousal space, including mildly aversive cues, in order to more fully capture approach–avoidance dynamics.

Finally, although the use of virtual reality provides ecological validity, the generalizability of the findings to real purchasing contexts requires further validation. Field studies are necessary to confirm the transfer of laboratory and VR-based results to real retail environments, where factors such as crowding, competing scents, and ambient noise may interact with sensory design. Future research should also extend recruitment to larger and more diverse populations, examining potential moderating factors such as olfactory sensitivity, age, or trait anxiety. Ultimately, the utility of neurophysiological measures such as HRV for managerial decision-making will be strengthened by directly linking physiological responses to commercial outcomes, thereby providing a robust basis for evidence-based design strategies in the retail environment.

Our findings highlight the importance of considering both subjective experiences and objective physiological measures when evaluating the impact of multisensory retail environments. Future research should explore these dynamics further, particularly focusing on how the interactions in different sensory combinations might influence emotional states and physiological responses over time. This could provide deeper insights into optimizing retail environments for enhanced consumer wellbeing.

## 5 Conclusion

In this study, we presented a scientific rather than an intuitive method for selecting sensory stimuli for the zones of a retail space. The described methods, technologies, and data analysis offer significant practical value for businesses that often face the challenge of positioning their products, spaces, or brands using sensory stimuli. The use of neurophysiological tools and algorithms allowed us to propose objective markers for assessing the valence of consumers’ emotional states in different multisensory environments. The valence markers of emotions are indicative of approach and avoidance behaviors, which directly characterize consumers’ desire to interact with a brand.

## Data Availability

The raw data supporting the conclusions of this article will be made available by the authors, without undue reservation.

## Footnotes

^1^ https://enjoysurvey.ru/
